# Comparative study between the Hybrid Capture II test and PCR based assay for the detection of human papillomavirus DNA in oral submucous fibrosis and oral squamous cell carcinoma

**DOI:** 10.1186/1743-422X-7-253

**Published:** 2010-09-23

**Authors:** Ajay Kumar Chaudhary, Shruti Pandya, Ravi Mehrotra, Alok C Bharti, Mangal Singh, Mamta Singh

**Affiliations:** 1 Centre for Biotechnology, University of Allahabad, India; 2 Department of Pathology, Moti Lal Nehru Medical College, Allahabad, India; 3 Department of Otorhinolaryngology, Moti Lal Nehru Medical College, Allahabad, India; 4 Division of Molecular Oncology, Institute of Cytology and Preventive Oncology (ICPO), NOIDA, India

## Abstract

**Background:**

Oral malignancy is a major global health problem. Besides the main risk factors of tobacco, smoking and alcohol, infection by human papillomavirus (HPV) and genetic alterations are likely to play an important role in these lesions. The purpose of this study was to compare the efficacy of HC-II assay and PCR for the detection of specific HPV type (HPV 16 E6) in OSMF and OSCC cases as well as find out the prevalence of the high risk HPV (HR-HPV) in these lesions.

**Methods and materials:**

Four hundred and thirty patients of the potentially malignant and malignant oral lesions were taken from the Department of Otorhinolaryngology, Moti Lal Nehru Medical College, Allahabad, India from Sept 2007-March 2010. Of which 208 cases were oral submucous fibrosis (OSMF) and 222 cases were oral squamous cell carcinoma (OSCC). The HC-II assay and PCR were used for the detection of HR-HPV DNA.

**Result:**

The overall prevalence of HR-HPV 16 E6 DNA positivity was nearly 26% by PCR and 27.4% by the HC-II assay in case of potentially malignant disorder of the oral lesions such as OSMF. However, in case of malignant oral lesions such as OSCC, 32.4% HPV 16 E6 positive by PCR and 31.4% by the HC-II assay. In case of OSMF, the two test gave concordant result for 42 positive samples and 154 negative samples, with an overall level of agreement of 85.4% (Cohen's kappa = 66.83%, 95% CI 0.553-0.783). The sensitivity and specificity of the test were 73.7% and 92.05% (p < 0.00). In case of OSCC, the two test gave concordant result for 61 positive samples and 152 negative samples, with an overall level of agreement of 88.3% (Cohen's kappa = 79.29, 95% CI 0.769-0.939) and the sensitivity and specificity of the test were 87.14% and 92.76% (p < 0.00).

**Conclusion:**

This study concluded that slight difference was found between the positivity rate of HR-HPV infection detected by the HC-II and PCR assay in OSMF and OSCC cases and the HC II assay seemed to have better sensitivity in case of OSCC.

## Background

Oral malignancy is a major global health problem and it constitutes the sixth most common malignancy. More than 90% of these malignancies representing a squamous cell carcinoma (SCC), which are often preceded by pre-existing oral lesions termed as potentially malignant disorders of the oral mucosa such as oral sub mucous fibrosis (OSMF) [1]. OSMF occurs most commonly in South East Asia, but many cases, it has been reported worldwide, in countries like China, UK, Kenya, Saudi Arabia, Pakistan and other parts of the world [2]. In India, about 5 millions people suffer from this disease [3].

Mehrotra et al reported that potentially malignant and malignant disorders of the oral mucosa were widespread in the patients visiting in the Medical College and SRN hospital, Allahabad and suggested that OSMF constituted the highest number of the patients in the potentially malignant group while in case of malignant group, OSCC was most prevalent in this region [4]. The habits of chewing tobacco and areca nut with or without betal quid are rampant in this area. Besides the main risk factors of tobacco, smoking and alcohol, infection by human papillomavirus (HPV) and genetic alterations are likely to play an important role in these lesions [5]. Oncogenic HPVs are a main causative agent for cervical cancer, but the role of HPV infection in OSMF and OSCC is less established.

Human papillomavirus is about 55 nm in diameter. It has a single circular double stranded DNA molecule and belongs to the family papillomaviridae. Its genome is made up of 7,200-8,000 base pairs with a molecular weight of 5.2 × 10^6 ^D. Molecular evidences also provide support to the role of high risk HPV, particularly HPV-16, in the pathogenesis of OSCC of the head and neck [6]. Kreimer et al reported that genomic DNA of oncogenic HPV has been detected approximately 26% of all OSCC of the head and neck worldwide [7] but the most accurate and consistent study for OSMF and OSCC, in which viral integration and the expression of viral oncogenes (E6 and E7) have been shown [8].

HPV detection in potentially malignant and malignant oral squamous cell carcinoma showed many discrepancies. Several studies reported the presence of HPV-DNA within these lesions with variable frequency. HPV16 and 18 genotypes were the most frequently found viruses in these lesions. Bouda et al suggested that high risk HPV E6/E7 transcripts and viral integration have also been detected in head and neck squamous cell carcinoma (HNSCC). Transcriptionally active HR-HPVs, particularly HPV-16 are found in a subset of HNSCC. HPV16-associated carcinogenesis is mediated by expression of the viral E6 and E7 oncoproteins, which cause deregulation of the cell cycle by inactivating p53 and pRb respectively [9]. Integration often disrupts the integrity and expression of the E1 and E2 open reading frames, which may affect the transcription of E6 and E7 genes [10-13]. In HPV-16 and HPV-18, the E2 proteins are active in virus proliferation, it control E6-E7 gene expression and are necessary for episomal virus production [10]. Specific viral genes (E6 and E7) from HPV types 16, 18, and 33 act as oncogenes [14,15].

Recentaly, a second-generation assay with improved diagnostic sensitivity has been developed known as hybrid capture II test (HC-II) and approved by the US food and drug administration (FDA). The performance characteristics of HC-II assay and the PCR for detection of HPV DNA have been compared in cervical lesions and results shown variation. [16-19]. Many testing techniques used to identify the prevalence rate of cervical as well as oral associated HPV infection have been developed but specific HPV testing may not be proper as a primary screening tool due to lack of specificity and sensitivity of the test. Newly developed molecular techniques have significantly assisted to indentifying the association of HR-HPV types with these oral lesions. The purpose of this study was to compare the efficacy of HC-II assay and PCR for the detection of specific HPV type (HPV 16 E6) in OSMF and OSCC cases as well as find out the prevalence rate of the HR-HPV in these lesions.

## Materials and methods

### Clinical data collection and sample collection

**Figure 1:**
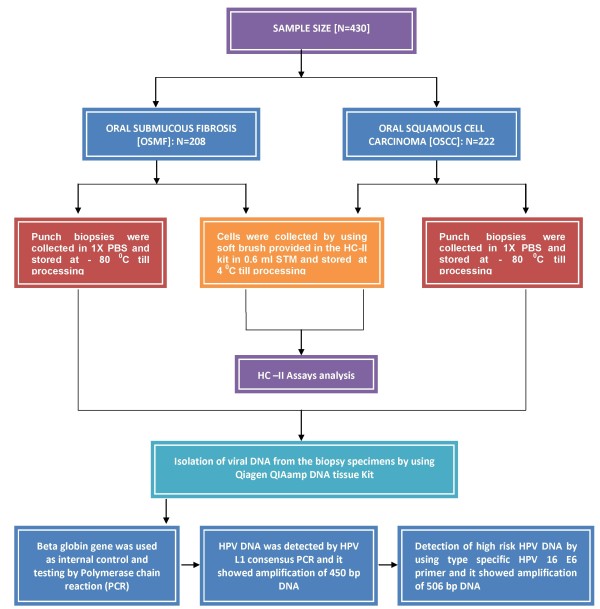
**Figure 1 illustrated the oral sub mucous fibrosis (OSMF) and oral squamous cell carcinoma (OSCC) specimen collection and HPV DNA testing methods**.

Four hundred and thirty patients of the OSMF and OSCC cases were taken from the Department of Pathology & Department of Otorhinolaryngology, Moti Lal Nehru Medical College, Allahabad, India from Sept 2007-March 2010, in a random manner after obtaining consent from the institutional ethical committee. Of which 208 cases were OSMF and 222 cases were OSCC. Detailed demographic information of each patient, including the patient's age, sex, addiction habits was obtained. Emphasis was given on their addiction habits. Usually those patients were considered who had no previous history of treatment for HPV infection. Figure 1 illustrating the oral sample collection and HPV testing strategy [Figure 1].

Detailed clinical examination of each patient was done to assess the site, size and type of lesions. For confirmation of the clinical diagnosis, histopathological examination was carried out. Patients with OSMF were included in this study and lesions with an abnormal epithelial surface like erythroplakia, leukoplakia and submucosal lesions including hemangiomas, mucoceles, papilloma, aphthous ulcers, melanoplakia and fibromas were excluded due to less number. A punch biopsy was performed as per standard protocol and the tissue was processed by paraffin embedding 2-3 micrometer thick sections were cut and stained by haematoxylin and eosin (H and E). Biopsies, which were inadequate on histopathology, were excluded from the study.

Histopathological examination was done and results were recorded according to the traditional grading of OSMF by Pindborg and Sirsat [20]. All specimens were examined independently by 2 different histopathologists in a double blind fashion. If there were any discrepancies, a third opinion was obtained. Two similar opinions constituted the final diagnosis.

Samples were collected from the suspicious lesions of the oral cavity by a soft brush provided in the kit. Gentle rolling strokes were made over the affected area as per the manufacturer's instructions and samples were collected in the sample collection tubes, stored at 4°C till the HC-II test was performed and also biopsies of the same site were taken in 1 × phosphate buffer saline (PBS) solution for the isolation of HPV-DNA.

#### Detection of 13 type high-risk HPV (HR-HPV) by hybrid capture-II (HC-II) assay

Second-generation commercial HR-HPV test, the HC II assay *Digene^® ^(manufacturer), a FDA approved test for clinical diagnostics was utilized. Detection of HPV DNA was carried out in the presence of a probe which consist the 13 high risk HPV (HR-HPV) types (16, 18, 31, 33, 35, 39, 45, 51, 52, 56, 58, 59 and 68). This technique is a nucleic acid hybridization assay with signal amplification that utilizes micro-plate chemiluminiscent detection.

#### Procedure of the HC-II test

First, double stranded DNA was denatured by using a strong alkaline denaturation solution and converted into single stranded DNA (ssDNA). This ssDNA was then hybridized in-solution to a cocktail of specific 13 HR- HPV RNA probes. The resultant DNA-RNA hybrids are captured onto the surface of a microwell plate coated with specific antibodies for DNA-RNA hybrids. The immobilized hybrids are then reacted with alkaline phosphatase conjugated antibody and detected by cleavage of the chemiluminescent substrate. The emitted light was measured as relative light unit (RLU) in a luminometer (DML 2000, Digene^®^). The intensity of the light was proportional to the amount of target DNA in the sample.

#### Estimation of HPV viral load

Ratio of relative light unit (RLU) of the sample/mean of RLU of three positive controls (PC) were taken as an estimate of approximate viral load which relates to the index of the intensity of HPV infection. This ratio of any specimen represents empirically a relative measure of the viral load in it. Cut off value (RLU of specimen/mean RLU of PC) was 1.0 pg/ml of sample.

#### Interpretation of Cut off value (RLU of specimen/mean RLU of PC)

Cut-off ratio of 0 to 0.99 is negative for HR HPV; Cut-off ratio greater than 1.0 positive for HR HPV. The cut off ratio at 1.0 correspond to viral DNA load of 5,000 copies/ml of 1pg/ml at a threshold of finding a clinical disease or prognosis of an OSMF and OSCC lesions.

### DNA extraction and diagnosis of high risk HPV infection by polymerase chain reaction

High molecular weight genomic DNA was extracted from oral biopsies of patients by using the Qiagen QIAamp DNA tissue Kit (Qiagen Inc. USA) The extracted genomic DNA was quantified and checked for purity spectrophotometrically (Spectro UV-Vis Double Beam PC, UVD Model 2950 LABOMED, Inc. CA, USA) Ethidium bromide (EtBr) stained 0.8% agrose gel electrophoresis was used to confirm presence of DNA in samples.

### Detection of HPV DNA

**Figure 2:**
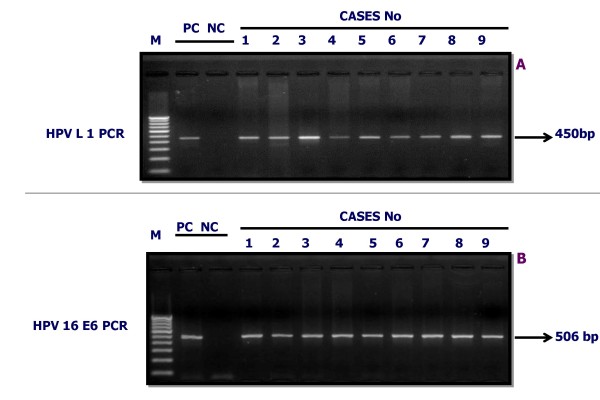
**Detection of HPV infection by PCR in OSMF and OSCC cases**. Gel figure A represent the ethidium bromide-staining in 2% agarose gel and showing presence of HPV infection in OSMF & OSCC cases with an amplicon of L1 consensus (450 bp) and Gel figure B represent the amplicon of HPV 16 E6 (506 bp). PC is positive control DNA, NC is negative control DNA, Lanes 1 to 9 are DNA samples from OSMF and OSCC cases, M = 100 bp molecular weight marker.

PCR amplification was performed using the consensus degenerate primers (L1 gene) [MY09: 5'-GCM CAG GGW CAT AAY AAT GG-3', MY11:5'-CGT CCM ARR GGA WAC TGA TC-3' where M = A/C; W = A/T; Y = C/T; R = A/G) with an amplicon size 450 bp, as described earlier [21]. Further typing of high risk HPV types 16 E6 was done by type-specific forward primer (FP) 5'-GAA ACC GGT TAG TAG TAT AAA AGC AGA C-3' and reverse primer (RP) 5'-AGC TGG GTT TCT CTA CGT GTT CT-3' with an amplicon size 506 bp [Figure 2]. β-globin gene primer was used as internal control, FP 5'-GAA GAG CCA AGG ACA GGT AC-3' and RP 5'-CAA CTT CAT CCA CGT TAC ACC-3', with an amplicon size 268 bp. HPLC purified primers were custom-synthesized by Metabion GmbH, Germany.

PCR was performed in a 25 μL reaction mixture containing 50-100 ng DNA, 10 mM Tris-HCl (pH 8.4), 50 mM KCl, 1.5 mM MgCl_2_, 100 μM of each dNTPs (dATP, dGTP, dCTP, dTTP) (Fermentas Inc.USA), 10 pmoles of oligonucleotide primers and 0.5U Taq DNA polymerase (Banglore Genei Pvt.Ltd.,India). The thermal cycler (PTC-100 MJ research GMI, Inc, Minnesota, USA) used, where an initial denaturation at 94°C for 5 min followed by 34 cycles of denaturation at 94°C for 30 s, annealing at 55°C for 30 s and extension at 72°C for 1 min, which was extended for 5 min at the final cycle.

### Statistical analysis

Level of agreement was calculated by both absolute agreement and Cohen's kappa statistic (k), a measure of the agreement between two methods in excess of that due to chance [22]. Proportions were compared with exact p values for the Pearson chi-square test. Statistical significance was achieved when the p value of the all test was <0.05. The odds ratio (OR) and 95% confidence interval (CI) were calculated using simple interactive statistical analysis (SISA) software package http://www.quantitativeskills.com/sisa/index.htm.

## Result

**Figure 3:**
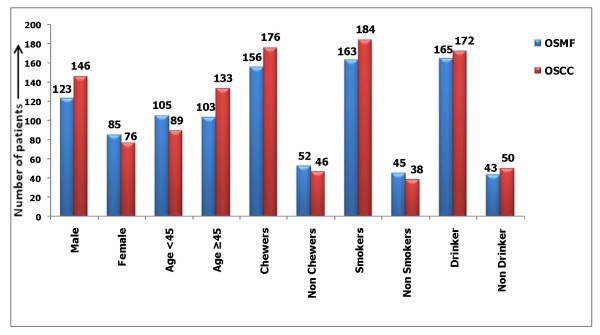
**Demographic distribution of the patients in OSMF and OSCC cases**.

Four hundred thirty cases (average age 58.0 ± 12.6 years) were incorporated in this study, of which 208 patients suffered from potentially malignant disorder of the oral cavity such as OSMF (123; 59.13% males and 85; 40.87% females) and 222 OSCC patients (146; 65.76% males and 76; 34.23% females). On the basis of demographic distribution of the patients with 208 cases of the OSMF, of which 105 (50.48%) and 103 (49.52%) cases were below and above the age of 45 years respectively while in 222 OSCC cases, 89 (40.1%) and 133 (59.9%) cases were below and above the age of 45 years respectively. In case of OSMF, 156 (75%), 163 (78.36%) and 165 (79.32%) subjects were addicted with tobacco chewing, cigarette smoking and alcohol drinking respectively while 52 (25%), 45 (21.63%) and 43 (20.67%) subjects were never taken any types of addiction. In case of OSCC, 176 (79.27%), 184 (82.88%) and 172 (77.47%) subjects were addicted with tobacco chewing, cigarette smoking and alcohol drinking respectively while 46 (20.72%), 38 (17.11%) and 50 (22.52%) subjects were never taken any types of addiction [Figure 3].

**Figure 4:**
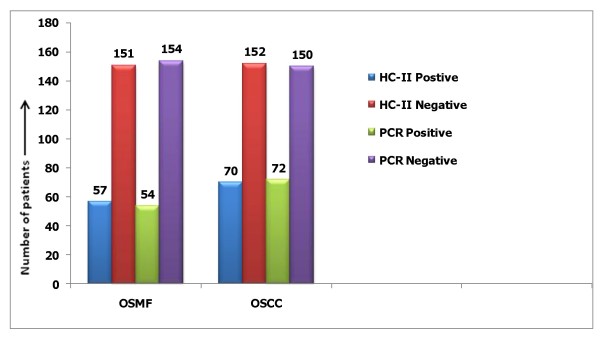
**Prevalence rate of HPV infection in OSMF ad OSCC cases by HC-II assay and PCR detection**.

Total 208 cases of OSMF, 57 (27.4%) were HR-HPV DNA positivite and 151 (72.59%) cases were negative while in 222 cases of OSCC, 70 (31.53%) were positive and 152 (68.47%) subjects were negative by HC-II assay. These sample were again tested by the type specific HPV 16 E6 with the help of PCR. The positivity rate of HPV 16 E6 DNA was 54 (25.96%) in OSMF and 72 (32.43%) in OSCC cases while 154 (74.03%) and 150 (67.56%) cases were negative respectively [Figure 4].

Table 1 summarizes the results of HPV DNA detection by the two tests in the following categories such as gender, age, addiction habits as well as location of the tumors. In OSMF cases, 42 subjects were positive, of which 28 (66.6%) males and 14 (33.3%) females were positive while 139 subjects were negative, of which 77 (55.4%) males and 62 (44.6%) females were negative by the HR-HC-II assay as well as type specific PCR. In case of OSCC, 61 subjects were positive, of which 42 (68.8%) males and 19 (31.1%) females were positive while 141subjects were negative of which 91 (64.5%) males and 50 (35.5%) females were negative by both the test HC-II and PCR assay.

**Table 1:** HPV detection by HC-II assay and PCR in OSMF and OSCC patients in the following categories.

Variables	OSMF cases (n = 208)				OSCC cases (n = 222)			
	
	PCR+ve, HC-II+ve (N = 42)	PCR+ve, HC II-ve (N = 12)	PCR-ve, HC-II+ve (N = 15)	PCR-ve, HC-II-ve (N = 139)	PCR+ve, HC-II+ve (N = 61)	PCR+ve, HC II-ve (N = 11)	PCR-ve, HC-II+ve (N = 09)	PCR-ve, HC-II-ve (N = 141)
**Gender**								
**Male**	28 (66.6%)	07 (58.3%)	11 (73.3%)	77 (55.4%)	42 (68.8%)	07 (63.6%)	06 (66.7%)	91 (64.5%)
**Female**	14 (33.3%)	05 (41.7%)	04 (26.7%)	62 (44.6%)	19 (31.1%)	04 (36.4%)	03 (33.3%)	50 (35.5%)

**Age (Years)**								
**< 45**	26 (61.9%)	04 (33.3%)	07 (46.7%)	68 (48.9%)	39 (63.9%)	05 (45.5%)	05 (55.5%)	40 (28.4%)
**≥45**	16 (38.1%)	08 (66.6%)	08 (53.3%)	71 (51.1%)	22 (36.1%)	06 (54.5%)	04 (44.4%)	101(71.6%)

**Tobacco**								
**Chewing Status**								
**Chewers**	38 (90.5%)	10 (83.3%)	12 (80.0%)	96 (69.1%)	52 (85.2%)	08 (72.7%)	07 (77.8%)	109(77.3%)
**Non Chewer**	04 (9.5%)	02 (16.6%)	03 (20.0%)	43 (30.9%)	09 (14.7%)	03 (27.3%)	02 (22.2%)	32 (22.7%)

**Smoking Status**								
**Smokers**	35 (83.3%)	08 (66.6%)	09 (60.0%)	111(79.8%)	51 (83.6%)	07 (63.6%)	05 (55.5%)	121(85.8%)
**Non smokers**	07 (16.6%)	04 (33.3%)	06 (40.0%)	28 (20.1%)	10 (16.4%)	04 (36.4%)	04 (44.4%)	20 (14.2%)

**Alcohol**								
**Drinker**	40 (95.2%)	08 (66.6%)	12 (80.0%)	105(75.5%)	40 (65.6%)	05 (45.4%)	07 (77.8%)	120 (85.1%)
**Drinker**	02 (4.7%)	04 (33.3%)	03 (20.0%)	34 (24.5%)	21 (34.4%)	06 (54.5%)	02 (22.2%)	
**Non Drinker**								21 (14.9%)

**Location**								
**Oral Cavity**	18 (42.8%)	05 (41.7%)	06 (40.0%)	68 (48.9%)	32 (52.5%)	05 (45.4%)	03 (33.3%)	72 (51.1%)
**Tongue**	12 (28.6%)	03 (25.0%)	04 (26.6%)	13 (9.4%)	12 (19.7%)	03 (27.3%)	04 (44.4%)	21 (14.9%)
**Lip**	09 (21.4%)	02 (16.6%)	04 (26.6%)	28 (20.1%)	08 (13.1%)	02 (18.2%)	02 (22.2%)	20 (14.2%)
**Hard & soft palate**	03 (7.1%)	02 (16.6%)	01 (6.7%)	30 (21.6%)	09 (14.7%)	01 (9.1%)	00 (0.0%)	28 (19.8%)

On the basis of addiction habits, in OSMF cases, 42 were positive by the HR-HC-II assay as well as type specific PCR. Of which, 38/42 (90.5%) tobacco chewers, 35/42 (83.3%) cigarette smokers, 40/42 (95.2%) alcohol drinkers and 04/42 (9.5%) non chewers, 07/42 (9.5%) non smokers and 02/42 (4.7%) non drinkers were HPV positive. While 139 were negative by both test. Of which, 96/139 (69.1%) tobacco chewers, 111/139 (79.8%) cigarette smokers, 105/139 (75.5%) alcohol drinkers and 43 (30.9%) non chewers, 28/139 (20.1%) non smokers and 34/139 (24.5%) non drinkers were negative.

In case of OSCC, 61 were positive by the HR-HC-II assay as well as type specific PCR. Of which, 52/61 (85.2%) tobacco chewers, 51/61 (83.6%) cigarette smokers, 40/61 (65.6%) alcohol drinkers and 09/61 (14.7%) non chewers, 10/61 (16.4%) non smokers, 21 (34.4%) non drinkers were positive. While 141 cases were negative by the both test. Of which, 109/141 (77.3%) tobacco chewers, 121/141 (85.8%) cigarette smokers, 120/141 (85.1%) alcohol drinkers and 32/141 (22.7%) non chewers, 20/141 (14.2%) non smokers, 21/141 (14.9%) non drinkers were negative.

On the basis of location of the lesions in potentially malignant OSMF of the oral cavity, out of 42 positive cases, 18 (42.8%) oral cavity, 12 (28.6%) tongue, 09 (21.4%) lip, 03 (70.1%) hard & soft palate were positive while out of 139 negative cases, 68 (48.9%) oral cavity, 13 (9.4%) tongue, 28 (20.1%) lip, 30(21.6%) hard & soft palate were HPV negative by the both the test HR-HC-II assay as well as type specific PCR. In case of OSCC, out of 61 positive cases, 32 (52.5%) oral cavity, 12 (19.7%) tongue, 08 (13.1%) lip, 09 (14.7%) hard & soft palate were positive while out of 141 negative cases, 72 (51.1%) oral cavity, 21 (14.9%) tongue, 20 (14.2%) lip, 29 (19.8%) hard & soft palate were HPV negative by the both the test HR-HC-II assay as well as type specific PCR [Table 1 ].

Clinico-pathological diagnosis of the potentially malignant disorders (OSMF) of oral cavity, out of 42 positive cases, 5 (11.9%) grade I, 09 (21.42%) grade II, 11 (26.12%) grade III , 17 (40.47%) grade IV were HPV positive while out of 139 negative cases, 18 (12.94%) grade I, 29 (18.7%) grade II, 37 (26.61%) grade III , 55 (39.56%) grade IV were HPV negative by the both the test [Table 2]. In case of malignant oral lesions (OSCC), according to TNM grading criteria, 28/80 (35%) T1-2 and 33/142 (23.23%) T3-4 sample were positive and in lymph node (N) category 21/66 (31.8%) N0 and 40/156 (2.56%) N1-3 were positive for both the test. While 45/80 (56.25%) cases of T1-2 category, 96/142 (67.6%) cases of T3-4 were HPV negative and 42/66 (63.63%) cases of N0, 99/156 (63.46%) of N1-3were HPV negative by both test [Table 3].

**Table 2:** HPV detection by HC-II assay and PCR Method in Clinico-pathogical parameter (*OSMF grading according to Pindborg and T and N category according to UICC classification*) in OSMF cases.

Clinico-pathogical Diagnosis	No (%) HPV detection by HC-II assay and PCR Method			
	
	**PCR+ve**, HC-II+ve [n = 42]	**PCR+ve**, HC II-ve [n = 12]	**PCR-ve**, HC-II+ve [n = 15]	**PCR-ve**, HC-II-ve [n = 139]
**Potentially malignant oral lesions: Oral sub mucous fibrosis (OSMF), n = 208 cases**				

**Grade-I (26)**	5 (11.9%)	1 (8.33%)	2 (13.33%)	18 (12.94%)

**Grade-II (43)**	9 (21.42%)	3 (25.0%)	2 (13.33%)	29 (18.9%)

**Grade-II (55)**	11 (26.12%)	3 (25.0%)	4 (26.67%)	37 (22.61%)

**Grade IV (84)**	17 (40.47%)	5 (41.67%)	7 (46.6%)	55 (39.56%)

**Table 3:** HPV detection by HC-II assay and PCR Method in Clinico-pathogical parameter (*OSMF grading according to Pindborg and T and N category according to UICC classification*) in OSCC cases.

Clinico-pathogical Diagnosis	No (%) HPV detection by HC-II assay and PCR Method			
	
	**PCR+ve**, HC-II+ve [n = 61]	**PCR+ve**, HC II-ve [n = 11]	**PCR-ve**, HC-II+ve [n = 09]	**PCR-ve**, HC-II-ve [n = 141]
**Malignant oral lesions: oral squamous cell carcinoma (OSCC), n = 222 cases**				

**T category**				
**T1-2 (80)**	28 (35%)	4 (5%)	3 (3.75%)	45 (56.25%)
**T3-4 (142)**	33 (23.23%)	7 (4.92%)	6 (4.22%)	96 (67.6%)

**N category**				
**N0 (66)**	21 (31.81%)	2 (3.03%)	1 (1.51%)	42 (63.63%)
**N1-3 (156)**	40 (2.56%)	9 (5.76%)	8 (5.12%)	99 (63.46%)

In case of OSMF, the two tests gave concordant results for 42 HPV positive samples and 154 HPV negative samples, with an overall level of agreement of 85.4% (Cohen's kappa = 66.83). Among the samples with discrepant results, 15 were positive by the HC-II assay but negative by PCR, whereas 12 samples were PCR positive but negative by HC-II assay. The sensitivity and specificity of the test were 73.7% and 92.05% (p < 0.00) respectively [Table 4].

**Table 4:** Concordance of results of HC-II assay and PCR test for detection of HPV infection in oral sub mucous fibrosis (OSMF): a potentially malignant oral lesions^a^

Hybrid Capture-II (HC-II)	HPV-16 E6 PCR		Total
		
	HPV-16 E6 Positive	HPV-16 E6 Negative	
**HC-II Positive**	42 (77.78%)	15 (9.74%)	57 (100)

**HC-II Negative**	12 (22.22%)	139 (90.26%)	151 (100)

**Total**	54	154	208

**^a^*Cohen's kappa (λ) = ****66.83% *(95%CI *0.5533-0.7834); *Sensitivity = 73.68% (95% CI: 0.6034-0.8446); Specificity = 92.05% (95% CI: 0.8653-0.9583); Pearson χ^2 ^p < 0.00.

In case of OSCC, the two tests gave concordant results for 61 HPV positive samples and 152 HPV negative samples, with an overall level of agreement of 88.3% (Cohen's kappa = 79.29, 95% CI 0.706-0.879). Among the samples with discrepant results, 11 were positive by the HC-II assay but negative by PCR, whereas 9 samples were PCR positive but negative by HC-II assay. The sensitivity and specificity of the test were 87.14% and 92.76% (p < 0.00) respectively [Table 5].

**Table 5:** Concordance of results of HC-II assay and PCR test for detection of HPV infection in oral squamous cell carcinoma (OSCC): a malignant oral lesions^a^

Hybrid Capture-II (HC-II)	HPV-16 E6 PCR		Total
		
	HPV-16 E6 Positive	HPV-16 E6 Negative	
**HC-II Positive**	61 (84.72%)	11 (15.28%)	72 (100)

**HC-II Negative**	09 (6.00%)	141 (94.00%)	150 (100)

**Total**	70	152	222

**^a^*Cohen's Kappa (λ) = ****79.29 *(95%CI *0.7067-0.8792); *Sensitivity = 87.14% (95% CI: 0.7699-0.9395); Specificity = 92.76% (95% CI: 0.8742-0.9633); Pearson χ^2 ^p < 0.00.

## Discussion

In this study we compare the data obtained by two techniques, (HC-II & PCR assay) detecting HR HPV DNA in potentially malignant (OSMF) and malignant (OSCC) disorders of the oral cavity. Use of the same samples for both DNA tests was done to avoid bias. We examined the punch biopsies sample histopathologically and to find out the final diagnosis of the oral lesions. This approach acceptable to find out the positivity rate between HPV detection and oral lesions. We did not detect any type of low risk humanpapilloma virus in these lesions. The HC-II assay used for the detection of the presence of 13 types high risk HPV types (i.e, 16,18,31, 35,39,45,51, 52, 58, 59 and 68) and 18 low risk (i.e, 6,11,42,43 and 44) in cervical lesions [23,24]. As well as recentaly Mehrotra et al reported that the prevalence rate of HR HPV infection in potentially malignant lesions (OSMF) with 33/105 (31.42%) HR-HPV positivity by the HC-II test [25].

This study included 430 cases (average age 58.0 ± 12.6 years), of which 208 patients suffered from potentially malignant lesions such as OSMF (123; 59.13% males and 85; 40.87% females) and 222 OSCC patients (146; 65.76% males and 76; 34.23% females). The overall occurrence rate of HPV 16 E6 DNA positivity was nearly 26% by PCR and 27.4% by the HC-II test in case of OSMF. While in case of OSCC the HPV DNA positivity were 32.4% by the PCR and 31.5% by the HC-II test. Therefore, the study concluded that slight difference was found between the positivity rate of HR HPV infection of the both detection methods in OSMF as well as in OSCC cases. However, Kujan et al from United Kingdom, reported that all oral samples were negative for HR-HPV using the HC-II technique [26] while Vidal et al from Brazil [27], studied the role of HPV by this assay in oral carcinoma and reported 22.5% positivity of HR-HPV DNA by the same test. They also suggested that the detection of HPV by this technique not only helped to identify viral infections, but also correspond to find out the koilocytosis on exfoliative cytology and concluded the presence of HPV might also contribute to the development of cancer, but there are many other factors such as tobacco smoking, tobacco chewing and radiations that were consistently present in persons suffering from oral malignancies.

In this study we reported that in case of potentially malignancy oral lesions such as OSMF, the two tests (HC-II & PCR assay) concordant results with an overall level of agreement of 85.4% (Cohen kappa = 66.83, 95% CI = 0.553-0.736). The sensitivity and specificity of the test were 73.7% and 92.5% (p < 0.00) respectively. While in case of OSCC both the tests gave concordant result with an overall level of agreement of 88.3% (Cohen's kappa = 79.29, 95% CI = 0.706-0.879). The sensitivity and specificity of the test were 87.14% and 92.76% (p < 0.00) respectively. Therefore, we concluded that HC-II assay seemed to have better sensitivity in case of OSCC. Similar result was reported by the Kalama et al in case of cervical lesions [24]. To the best of our knowledge this is the first study to find out the occurrence rate as well as the comparative study between the HC-II assays and PCR in OSMF and OSCC cases. Investigation on the role of HPV markers could be rewarding in planning long-term strategies for prevention, diagnosis and possible cure of these conditions. There findings warrant further study with a larger number of patients. Evidence has showed that strong epidemiology data would provide additional support for a causal association between HR-HPV and oral lesions and might be guided future cancer prevention programs involving vaccination to oral HPV infection or screening in North Indian population.

## Conclusion

This study concluded that slight difference was found between the positivity rate of HR-HPV infection detected by the HC-II and PCR assay in OSMF and OSCC cases and the HC II assay seemed to have better sensitivity in case of OSCC.

## Abbreviations

(HPV): Human papillomavirus; (PCR): Polymerase chain reaction; (OSMF): Oral submucous fibrosis; (HNSCC): head and neck squamous cell carcinoma; (HC-II): Hybrid Capture; (OSCC): Oral squamous cell carcinoma.

## Competing interests

The authors declare that they have no competing interests.

## Authors' contributions

AKC and SP carried out the experimental work, analysis and drafted the manuscript. Mamta Singh conceived of the study, participated in its design and coordination as well as helped to draft the manuscript. MS, ACB and RM participated in coordination of the study. All authors read and approved the final manuscript.
